# A study on the association between smoking, periodontal inflammation and alveolar bone resorption in patients with chronic periodontitis based on biomarkers: a meta-analysis

**DOI:** 10.3389/fmed.2025.1666722

**Published:** 2026-01-05

**Authors:** Bing Li, Li Xia, Cui Liu, Qingqing Zhu, Zhe Chen

**Affiliations:** 1 Department of Stomatology, Central Medical Branch of PLA General Hospital, Beijing, China; 2 Department of Stomatology, Beijing Jishuitan Hospital, Capital Medical University, Beijing, China

**Keywords:** smoking, inflammatory response, periodontal tissue, alveolar bone resorption, chronic periodontitis

## Abstract

**Objective:**

To systematically evaluate the association between smoking and both periodontal inflammation and alveolar bone resorption in patients with chronic periodontitis.

**Methods:**

A computerized search was conducted using databases such as PubMed, Embase, Web of Science, and China National Knowledge Infrastructure to identify studies published between January 2005 and January 2023 that examined the relationship between smoking and both periodontal inflammation and alveolar bone resorption in chronic periodontitis. A total of 1,654 articles were initially retrieved. After applying the inclusion and exclusion criteria, 13 studies were included in the final analysis, comprising a total of 1,653 patients—893 in the study group and 760 in the control group. The study group included smoker chronic periodontitis patients, and the control group included non-smoker chronic periodontitis patients. Two independent researchers screened the literature and extracted relevant data. Meta-analysis was performed using RevMan 5.3 software.

**Results:**

Compared with the control group, the study group showed significantly higher levels of intercellular adhesion molecule 1 (mean difference [MD] = 135.93; 95% confidence interval [CI]: 48.91, 222.95; *p* = 0.002), high-mobility group box 1 (MD = 6.02; 95% CI: 1.30, 10.73; *p* = 0.01), interleukin-17 (MD = 94.62; 95% CI: 23.34, 165.90; *p* = 0.009), C-terminal telopeptide of type I collagen (MD = 14.56; 95% CI: 12.03, 17.09; *p* < 0.00001), receptor activator of nuclear factor kappa-B ligand (MD = 1.49; 95% CI: 0.85, 2.14; *p* < 0.00001) and matrix metalloproteinase 9 (MD = 27.15; 95% CI: 5.94, 48.37; *p* = 0.01), as well as lower levels of osteoprotegerin (MD = −1.52; 95% CI: −2.98, −0.06; *p* = 0.04). There were no significant differences in chitinase-3-like protein 1 levels (MD = 16.40; 95% CI: −3.84, 36.64; *p* = 0.11), procollagen type I N-terminal propeptide levels (MD = −4.35; 95% CI: −10.71, 2.02; *p* = 0.18) and N-terminal telopeptide of type I collagen levels (MD = 9.03; 95% CI: −6.75, 24.80; *p* = 0.26) in the gingival crevicular fluid between the two groups.

**Conclusion:**

Smoking exacerbates periodontal inflammation and promotes alveolar bone resorption in patients with chronic periodontitis.

## 1 Introduction

Chronic periodontitis is a very common periodontal disease worldwide, with a persistently high prevalence rate, which seriously affects people’s oral health as well as their overall health (1). Smoking, as a widespread and highly harmful lifestyle habit, has been recognized by numerous studies as one of the main factors contributing to the occurrence and development of chronic periodontal disease (2). Although numerous studies have focused on the association between smoking and periodontal diseases (3, 4), there is still no clear and definite conclusion regarding exactly how smoking affects the degree of periodontal inflammation and alveolar bone resorption in patients with chronic periodontitis.

On the one hand, there are significant inconsistencies among different studies in assessing the impact of smoking on the severity of periodontal inflammation in patients with chronic periodontitis. Some studies suggest that smoking significantly aggravates periodontal inflammation, manifested by swollen and bleeding gums, increased probing depth, and more severe attachment loss (5). However, other studies have reached completely different or even contradictory conclusions. Some research indicates that although smoking may affect the local environment of the periodontal tissues to some extent, in the quantitative assessment of the overall severity of periodontal inflammation, there is no such significant difference compared to non-smoking patients (6). The inconsistency of these research results may be caused by various factors such as differences in the selection of research samples, subtle differences in diagnostic criteria, and limitations of research methods. This inconsistency makes it difficult for clinicians to accurately determine the specific impact of smoking on periodontal inflammation when dealing with patients with chronic periodontal disease caused by smoking, thereby affecting the formulation of personalized treatment plans.

On the other hand, there are still many unresolved uncertainties regarding the impact of smoking on alveolar bone resorption in patients with chronic periodontitis. Some studies have emphasized that smoking accelerates the process of alveolar bone resorption, significantly increasing the risk of tooth loosening and loss (7). However, some studies suggest that the relationship between smoking and alveolar bone resorption is not a simple linear correlation; it may be influenced by the interaction of multiple other factors. For instance, factors such as the patient’s age, gender, overall health condition, and oral hygiene habits may all play a regulatory role in the effect of smoking on alveolar bone resorption (8). However, at present, there is a lack of in-depth and systematic research on how these factors specifically influence the relationship between smoking and alveolar bone resorption. This makes it impossible for us to precisely predict the development trend of alveolar bone resorption in patients with chronic periodontal disease due to smoking, nor can we formulate more effective prevention and treatment strategies to stop or slow down the resorption of alveolar bone.

The pathogenesis of chronic periodontitis is complex and involves various changes at the cellular and molecular levels (1). The progression of periodontal inflammation and the process of alveolar bone resorption do not occur independently but are finely regulated by a series of biological signaling molecules (9). These biomarkers, as key signaling molecules during the occurrence and development of the disease, can reflect the pathological and physiological state of the periodontal tissues from different perspectives (10). Therefore, using biomarker-based methods to study the association between smoking, periodontal inflammation, and alveolar bone resorption has unique advantages and significant importance.

This study carefully selected a series of representative biomarkers for analysis, including intercellular adhesion molecule 1 (ICAM-1), high-mobility group box 1 (HMGB1), interleukin-17 (IL-17), chitinase-3-like protein 1 (YKL-40), procollagen type I N-terminal propeptide (PINP), N-terminal telopeptide of type I collagen (NTX), C-terminal telopeptide of type I collagen (CTX), osteoprotegerin (OPG), receptor activator of nuclear factor kappa-B ligand (RANKL), and matrix metalloproteinase 9 (MMP-9). Among them, ICAM-1 plays a crucial role in the inflammatory response. It is involved in the adhesion process between white blood cells and vascular endothelial cells, and is an important mediator for the migration of inflammatory cells to the inflammatory site (11). In the periodontal tissues of patients with chronic periodontitis, the expression level of ICAM-1 may change due to the influence of smoking, thereby affecting the infiltration of inflammatory cells and the intensity of the inflammatory response (12). Therefore, it is selected as one of the indicators for evaluating the degree of periodontal inflammation. HMGB1 is a highly conserved nuclear protein that can be actively or passively released into the extracellular space under conditions of cell damage or stress (13). As an advanced inflammatory mediator, it participates in the occurrence and development of various inflammatory diseases (14). Smoking may induce the release of HMGB1 from periodontal tissue cells, exacerbating local inflammatory responses (15). Therefore, including it in the research scope is helpful for a deeper understanding of the mechanism of smoking’s impact on periodontal inflammation. IL-17 is an important pro-inflammatory cytokine, mainly secreted by Th17 cells, and plays a significant role in autoimmune diseases and chronic inflammatory disorders (16). In chronic periodontitis, IL-17 can promote the infiltration of inflammatory cells and the release of inflammatory mediators, thereby exacerbating the destruction of periodontal tissues (17). Smoking may affect the expression level of IL-17 and regulate the severity of periodontal inflammation (18). Therefore, detecting IL-17 can help reveal the intrinsic connection between smoking and periodontal inflammation. YKL-40 is a glycoprotein that is expressed at higher levels in various physiological and pathological processes such as inflammation, tissue repair, and tumor formation (19). In the periodontal tissues of patients with chronic periodontitis, the expression of YKL-40 may be related to the inflammatory response and alveolar bone resorption (20). Smoking may participate in the destruction process of periodontal tissues by influencing the expression of YKL-40 (21). Therefore, detecting YKL-40 is helpful for comprehensively evaluating the impact of smoking on chronic periodontitis. PINP, NTX and CTX are important indicators reflecting the state of bone metabolism (22). PINP is a product released during the synthesis of type I collagen, and its elevated level indicates active bone formation (23); NTX and CTX are products of the degradation of type I collagen, and their elevated levels reflect enhanced bone resorption (24). In patients with chronic periodontitis, alveolar bone resorption is an important pathological feature (25). Smoking may accelerate alveolar bone resorption by affecting the expression of bone metabolism-related indicators (26). Therefore, detecting these indicators is crucial for evaluating the impact of smoking on alveolar bone resorption. OPG and RANKL are key factors regulating bone metabolism. The balance between these two plays a decisive role in maintaining bone homeostasis (27). OPG can competitively bind to RANKL, inhibiting the differentiation and activation of osteoclasts, thereby suppressing bone resorption (28); while RANKL promotes the formation and activity of osteoclasts, enhancing bone resorption (29). Smoking may interfere with the balance of the OPG/RANKL system, affecting the process of alveolar bone resorption (30). Therefore, the detection of these two indicators is helpful for a deeper understanding of the mechanism of smoking in alveolar bone resorption. MMP-9 is a type of matrix metalloproteinase that can degrade various components in the extracellular matrix, such as collagen and gelatin (31). In chronic periodontitis, the excessive expression of MMP-9 can damage the structural integrity of periodontal tissues and promote alveolar bone resorption (32). Smoking may upregulate the expression of MMP-9, exacerbating the destruction of periodontal tissues (33). Therefore, detecting MMP-9 is of great significance for evaluating the impact of smoking on periodontal tissues. However, at present, the research on the expression changes of these biomarkers in patients with chronic periodontitis due to smoking and their specific relationships with periodontal inflammation and alveolar bone resorption is still insufficient. Different studies have differences in the detection methods of these biomarkers and the interpretation of the results, and there is a lack of research on the combined analysis of multiple biomarkers, making it difficult to comprehensively and accurately reflect the impact of smoking on the periodontal condition of patients with chronic periodontitis.

Therefore, this study aimed to systematically evaluate the relationship between smoking and periodontal inflammation and alveolar bone resorption in patients with chronic periodontitis through a meta-analysis. By comprehensively analyzing various biological markers mentioned above, this study intends to more comprehensively and deeply clarify the specific impact of smoking on the severity of periodontal inflammation and bone loss in affected individuals, providing new theoretical basis and ideas for the prevention, diagnosis, and treatment of chronic periodontitis.

## 2 Methods

### 2.1 Inclusion and exclusion criteria

The inclusion criteria were as follows: studies involving patients with chronic periodontitis; observational studies; availability of data on the association between smoking and both periodontal inflammation and alveolar bone resorption in patients with chronic periodontitis; and publication in non-Chinese literature.

Studies were excluded if they involved non-human subjects, were designed as case reports, reviews, expert opinions, or literature reviews, or if they did not provide data on the relationship between smoking and both periodontal inflammation and alveolar bone resorption in patients with chronic periodontitis.

### 2.2 Literature search strategy

Relevant literature databases, including PubMed, Embase, Web of Science, and China National Knowledge Infrastructure, were systematically searched using computer-based queries. Studies investigating the association between smoking and both periodontal inflammation and alveolar bone resorption in patients with chronic periodontitis were collected. The search included articles published between January 2005 and January 2023. Keywords such as “smoking,” “chronic periodontitis,” “inflammatory response,” and “alveolar bone resorption” were used, combined with Boolean operators (e.g., AND, OR) to refine the search strategy.

### 2.3 Literature screening and data extraction

The retrieved literature was screened based on the predefined inclusion and exclusion criteria. Initially, the titles and abstracts were reviewed to eliminate studies that were irrelevant to the research topic. Subsequently, the full texts of the remaining articles were assessed for eligibility according to the same criteria. Two independent researchers conducted the screening process, and any disagreements were resolved through discussion or consultation with a third-party expert. Data extraction forms were developed in line with the study objectives and research questions. Extracted data included basic study information (such as author, year of publication, and study type), participant characteristics, study design, main findings, and conclusions. Data extraction was performed independently by two investigators and cross-verified to ensure accuracy.

### 2.4 Literature quality assessment

In this study, the Newcastle–Ottawa scale (34) was used to assess the quality of the included studies. Each item on the scale was scored based on predefined criteria, with higher scores reflecting better study quality. The overall quality was then classified according to the total score: studies scoring 7–9 were considered high quality, those scoring 4–6 were considered moderate quality, and those scoring 0–3 were considered low quality.

### 2.5 Statistical analysis

Meta-analysis was conducted using RevMan 5.3 software. Heterogeneity among studies was evaluated using the Q and I^2^ statistics. The Q statistic, based on the chi-square test, was used to assess whether the observed differences were attributable to chance, whereas the I^2^ statistic quantified the degree of heterogeneity, ranging from 0 to 100%, with higher values indicating greater heterogeneity. A 95% confidence interval (CI) was used to estimate the precision of the odds ratios. The mean difference (MD), representing the relative difference between two values, was used to evaluate the effect of a given factor on disease progression, treatment outcomes, and other relevant measures.

## 3 Results

### 3.1 Literature screening results

**Figure 1:**
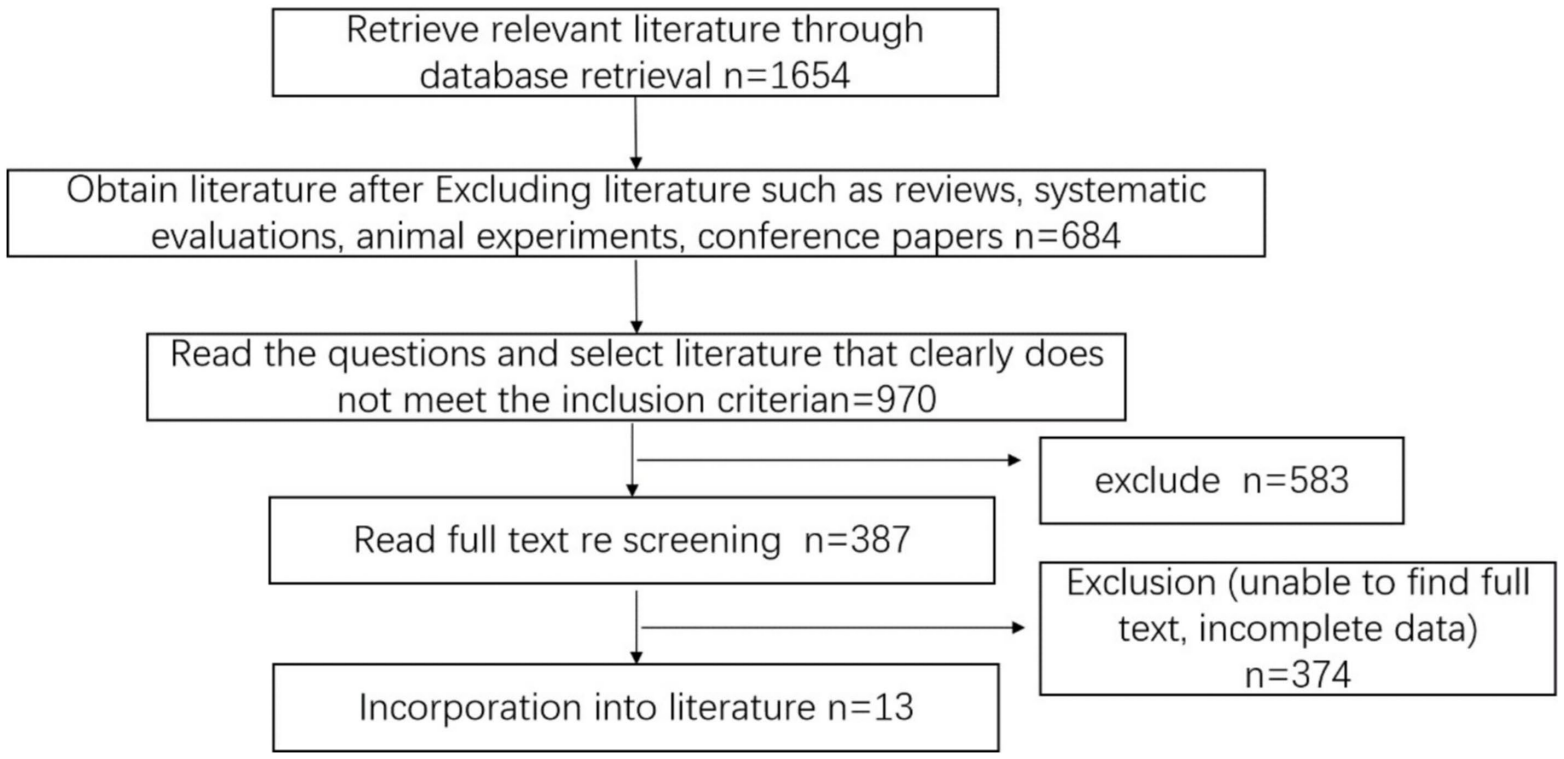
Literature screening flowchart.

Using the described search strategy, a total of 1,654 articles were initially retrieved. After applying the inclusion and exclusion criteria, 13 studies (35–47) were included in the final analysis, comprising a total of 1,653 patients—893 in the study group and 760 in the control group. The literature screening process is illustrated in Figure 1.

### 3.2 Basic characteristics and quality assessment of included studies

The basic characteristics and quality assessments of the included studies are presented in Table 1.

**Table 1:** Basic characteristics and quality assessments of included studies.

Author	Year	Study group	Control group	NOS score	Research indices
Li (39)	2018	49	25	7 points	①②③④⑤⑥⑦⑧⑨⑩
He (40)	2016	38	40	8 points	⑩
Bayan (41)	2022	32	42	9 points	③
Çiğdem (42)	2021	20	20	9 points	①⑤
Lappin (43)	2007	35	35	8 points	⑧⑨
Buduneli (44)	2008	5	7	8 points	⑧⑨
Buduneli (45)	2011	10	10	8 points	⑧⑨
Soo-Jeong (46)	2011	330	330	9 points	⑩
Ningombam (47)	2023	21	21	7 points	⑦
Lin (52)	2017	9	17	7 points	②
Alshahrani (53)	2021	15	15	8 points	②③
Zeynep (54)	2020	26	26	8 points	④
Che (55)	2015	303	172	8 points	⑥

① ICAM-1; ② HMGB1; ③ IL-17; ④ YKL-40; ⑤ PINP; ⑥ NTX; ⑦ CTX; ⑧ OPG; ⑨ RANKL; ⑩ MMP-9. NOS, Newcastle–Ottawa scale; ICAM-1, intercellular adhesion molecule 1; HMGB1, high-mobility group box 1; IL-17, interleukin-17; YKL-40, chitinase-3-like protein 1; PINP, procollagen type I N-terminal propeptide; NTX, N-terminal telopeptide of type I collagen; CTX, C-terminal telopeptide of type I collagen; OPG, osteoprotegerin; RANKL, receptor activator of nuclear factor kappa-B ligand; MMP-9, matrix metalloproteinase 9.

### 3.3 Meta-analysis results

#### 3.3.1 ICAM-1

**Figure 2:**
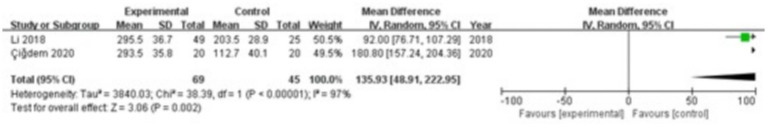
Forest plot of intercellular adhesion molecule 1 (ICAM-1) levels in gingival crevicular fluid in the two groups.

Two studies were included, comprising 69 patients in the study group and 45 in the control group. The heterogeneity test showed I^2^ = 97% and *p* < 0.00001, indicating substantial heterogeneity between the groups; therefore, a random-effects model was applied for the meta-analysis. The results demonstrated that ICAM-1 levels in the gingival crevicular fluid were significantly higher in the study group compared with the control group (MD = 135.93; 95% CI: 48.91, 222.95; *p* = 0.002), as shown in Figure 2.

#### 3.3.2 HMGB1

**Figure 3:**
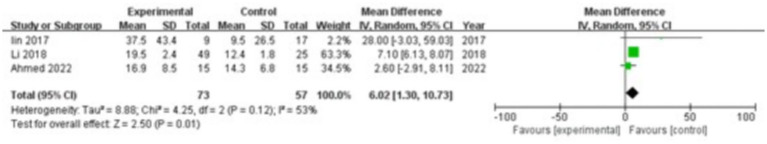
Forest plot of high-mobility group box 1 (HMGB1) levels in gingival crevicular fluid in the two groups.

Three studies were included, comprising 73 patients in the study group and 57 in the control group. The heterogeneity test showed I^2^ = 53% and *p* = 0.12, indicating moderate heterogeneity between the groups; therefore, a random-effects model was used for the meta-analysis. The results revealed that HMGB1 levels in the gingival crevicular fluid were significantly higher in the study group compared with the control group (MD = 6.02; 95% CI: 1.30, 10.73; *p* = 0.01), as shown in Figure 3.

#### 3.3.3 IL-17

**Figure 4:**
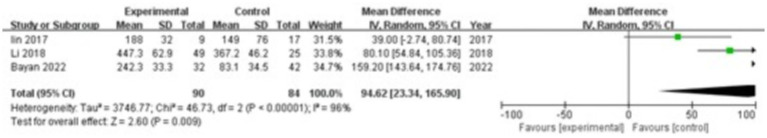
Forest plot of interleukin-17 (IL-17) levels in gingival crevicular fluid in the two groups.

Three studies were included, comprising 90 patients in the study group and 84 in the control group. The heterogeneity test showed I^2^ = 96% and *p* < 0.00001, indicating significant heterogeneity between the groups; therefore, a random-effects model was applied for the meta-analysis. The results demonstrated that IL-17 levels in the gingival crevicular fluid were significantly higher in the study group compared with the control group (MD = 94.62; 95% CI: 23.34, 165.90; *p* = 0.009), as shown in Figure 4.

#### 3.3.4 YKL-40

**Figure 5:**
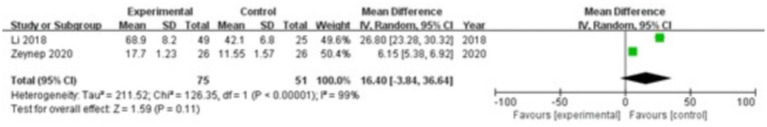
Forest plot of chitinase-3-like protein 1 (YKL-40) levels in gingival crevicular fluid in the two groups.

Two studies were included, comprising 75 patients in the study group and 51 in the control group. The heterogeneity test showed I^2^ = 99% and *p* < 0.00001, indicating substantial heterogeneity between the groups; therefore, a random-effects model was used for the meta-analysis. The results showed that there were no significant differences in YKL-40 levels in the gingival crevicular fluid between the two groups (MD = 16.40; 95% CI: −3.84, 36.64; *p* = 0.11), as shown in Figure 5.

#### 3.3.5 PINP

**Figure 6:**
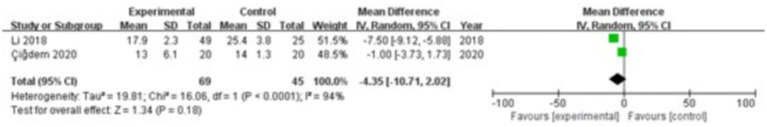
Forest plot of procollagen type I N-terminal propeptide (PINP) levels in gingival crevicular fluid in the two groups.

Two studies were included, comprising 69 patients in the study group and 45 in the control group. The heterogeneity test showed I^2^ = 94% and *p* < 0.0001, indicating considerable heterogeneity between the groups; therefore, a random-effects model was applied for the meta-analysis. The results revealed that there were no significant differences in PINP levels in the gingival crevicular fluid between the two groups (MD = −4.35; 95% CI: −10.71, 2.02; *p* = 0.18), as shown in Figure 6.

#### 3.3.6 NTX

**Figure 7:**
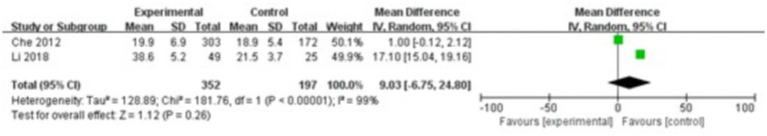
Forest plot of N-terminal telopeptide of type I collagen (NTX) levels in gingival crevicular fluid in the two groups.

Two studies were included, comprising 352 patients in the study group and 197 in the control group. The heterogeneity test showed I^2^ = 99% and *p* < 0.00001, indicating substantial heterogeneity between the groups; therefore, a random-effects model was used for the meta-analysis. The results demonstrated that there were no significant differences in NTX levels in the gingival crevicular fluid between the two groups (MD = 9.03; 95% CI: −6.75, 24.80; *p* = 0.26), as shown in Figure 7.

#### 3.3.7 CTX

**Figure 8:**
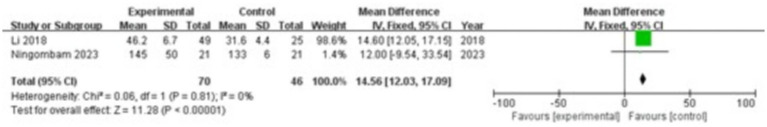
Forest plot of C-terminal telopeptide of type I collagen (CTX) levels in gingival crevicular fluid in the two groups.

Two studies were included, comprising 70 patients in the study group and 46 in the control group. The heterogeneity test showed I^2^ = 0% and *p* = 0.81, indicating no significant heterogeneity between the groups; therefore, a fixed-effect model was applied for the meta-analysis. The results showed that CTX levels in the gingival crevicular fluid were significantly higher in the study group compared with the control group (MD = 14.56; 95% CI: 12.03, 17.09; *p* < 0.00001), as shown in Figure 8.

#### 3.3.8 OPG

**Figure 9:**
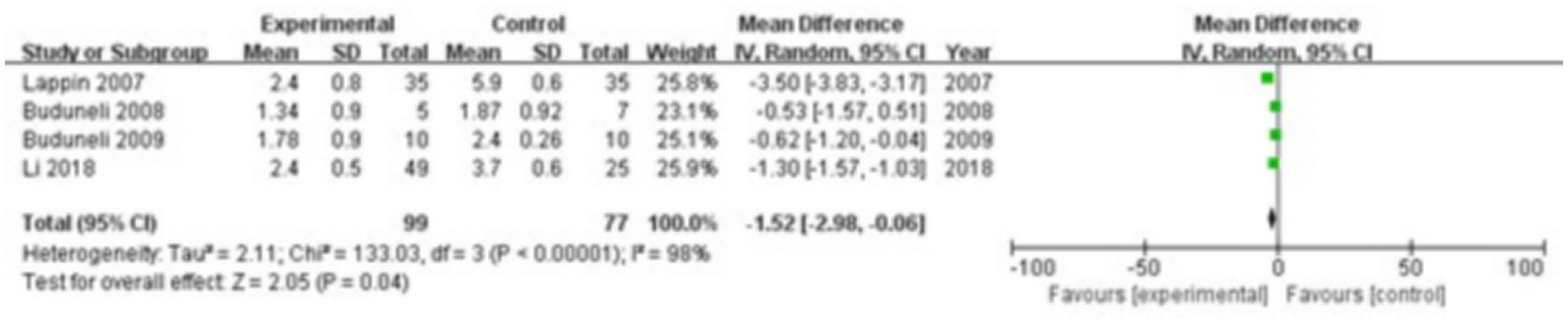
Forest plot of osteoprotegerin (OPG) levels in gingival crevicular fluid in the two groups.

Four studies were included, comprising 99 patients in the study group and 77 in the control group. The heterogeneity test showed I^2^ = 98% and *p* < 0.00001, indicating substantial heterogeneity between the groups; therefore, a random-effects model was used for the meta-analysis. The results revealed that OPG levels in the gingival crevicular fluid were significantly lower in the study group compared with the control group (MD = −1.52; 95% CI: −2.98, −0.06; *p* = 0.04), as shown in Figure 9.

#### 3.3.9 RANKL

**Figure 10:**
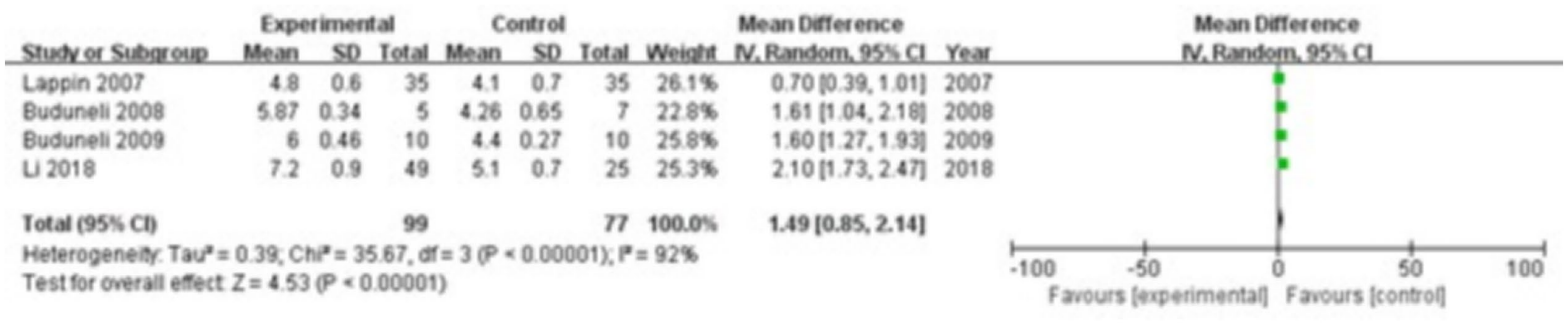
Forest plot of receptor activator of nuclear factor kappa-B ligand (RANKL) levels in gingival crevicular fluid in the two groups.

Four studies were included, comprising 99 patients in the study group and 77 in the control group. The heterogeneity test showed I^2^ = 92% and *p* < 0.00001, indicating considerable heterogeneity between the groups; therefore, a random-effects model was applied for the meta-analysis. The results demonstrated that RANKL levels in the gingival crevicular fluid were significantly higher in the study group compared with the control group (MD = 1.49; 95% CI: 0.85, 2.14; *p* < 0.00001), as shown in Figure 10.

#### 3.3.10 MMP-9

**Figure 11:**
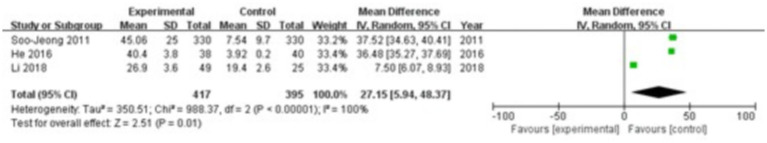
Forest plot of matrix metalloproteinase 9 (MMP-9) levels in gingival crevicular fluid in the two groups.

Three studies were included, comprising 417 patients in the study group and 395 in the control group. The heterogeneity test showed I^2^ = 100% and *p* < 0.00001, indicating significant heterogeneity between the groups; therefore, a random-effects model was used for the meta-analysis. The results showed that MMP-9 levels in the gingival crevicular fluid were significantly higher in the study group compared with the control group (MD = 27.15; 95% CI: 5.94, 48.37; *p* = 0.01), as shown in Figure 11.

## 4 Discussion

Smoking shows a strong association with periodontitis, with smokers facing a higher risk of developing the condition than non-smokers. By altering the composition of oral microbiota, impairing the clearance of pathogenic microorganisms, and suppressing the immune response, smoking promotes the onset and progression of periodontitis. However, the specific mechanisms through which smoking contributes to periodontitis remain unclear. Chronic periodontitis involves sustained activation of the periodontal inflammatory response, during which numerous inflammatory mediators are secreted into the gingival crevicular fluid. ICAM-1, a cell adhesion molecule, plays a key role in inflammation. Studies have shown elevated ICAM-1 levels in the gingival crevicular fluid of patients with periodontitis (48), with expression levels correlating with inflammation severity and tissue damage. HMGB1, a nuclear protein, contributes to inflammatory responses and immune regulation (49). IL-17, an inflammatory mediator, plays a critical role in periodontal inflammation. Evidence indicates that IL-17 expression increases in the gingival crevicular fluid of patients with periodontitis and correlates with both the severity of inflammation and the extent of tissue destruction (50). YKL-40, a quasi-inflammatory protein, contributes to both inflammation and tissue remodeling. Reports have documented elevated YKL-40 levels in the gingival crevicular fluid of patients with periodontitis, with correlations noted between its expression and the severity of inflammation, degree of tissue damage, and treatment outcomes (51). This meta-analysis revealed significantly higher levels of ICAM-1 (MD = 135.93; 95% CI: 48.91, 222.95; *p* = 0.002), HMGB1 (MD = 6.02; 95% CI: 1.30, 10.73; *p* = 0.01), and IL-17 (MD = 94.62; 95% CI: 23.34, 165.90; *p* = 0.009) in the gingival crevicular fluid of the smoking group compared with the control group. However, there were no significant differences in YKL-40 levels in the gingival crevicular fluid between the two groups (MD = 16.40; 95% CI: −3.84, 36.64; *p* = 0.11).

Periodontitis represents a major cause of alveolar bone resorption. The persistent inflammatory response associated with periodontitis contributes to the breakdown and loss of alveolar bone, ultimately leading to tooth mobility and loss. PINP, NTX, and CTX serve as biomarkers for assessing bone metabolism. Measuring these markers provides insight into bone metabolic status in patients with periodontitis. Previous studies have reported elevated levels of PINP, NTX, and CTX in patients with periodontitis compared with healthy individuals, suggesting that periodontitis may lead to abnormal bone metabolism (35, 36). This meta-analysis showed that there were no significant differences in PINP levels (MD = −4.35; 95% CI: −10.71, 2.02; *p* = 0.18) and NTX levels (MD = 9.03; 95% CI: −6.75, 24.80; *p* = 0.26) in the gingival crevicular fluid between the two groups. In contrast, CTX levels were significantly higher in the study group compared with the control group (MD = 14.56; 95% CI: 12.03, 17.09; *p* < 0.00001), suggesting that alveolar bone in patients with periodontitis may undergo excessive resorption and that smoking could further exacerbate this process.

The progression of periodontitis involves the release of inflammatory mediators and cytokines that contribute to alveolar bone resorption. The inflammatory response enhances both the formation and activity of osteoclasts, leading to increased bone breakdown. Osteoblasts, responsible for bone formation and repair, exhibit reduced activity in the inflammatory environment of periodontitis, as inflammatory mediators and chemical factors impair their function. This inhibition of bone repair further accelerates alveolar bone loss. Osteoclasts, the specialized cells that resorb bone, become more active under the influence of pro-inflammatory cytokines. These cells secrete bone-degrading enzymes, such as acid proteases and MMPs, which intensify the resorption of alveolar bone. Collagen, a primary structural component of alveolar bone, becomes a key target in this process. Inflammatory mediators and bone-degrading enzymes destroy collagen fibers and enhance collagen hydrolysis, further promoting bone loss. OPG serves as a critical regulator by inhibiting osteoclast formation and activity through competitive binding with RANKL (37). In contrast, MMP-9, a bone-degrading enzyme, facilitates collagen breakdown and contributes to alveolar bone resorption (38). This study found significantly lower OPG levels in the gingival crevicular fluid of the study group compared with the control group (MD = −1.52; 95% CI: −2.98, −0.06; *p* = 0.04), and significantly higher levels of RANKL (MD = 1.49; 95% CI: 0.85, 2.14; *p* < 0.00001) and MMP-9 (MD = 27.15; 95% CI: 5.94, 48.37; *p* = 0.01). These findings suggest an imbalance between bone-resorbing and bone-protective molecules in patients with periodontitis, and indicate that smoking may exacerbate this dysregulation, further promoting alveolar bone resorption.

When comparing the results of different studies regarding the expression of biomarkers, we did observe significant differences, which imposed certain limitations on the comprehensive analysis of the research outcomes. These differences might be caused by various factors. Firstly, the differences in the characteristics of the research subjects are an important factor. The patients included in different studies may vary significantly in terms of age, gender, race, severity of periodontal disease, and overall health status. Secondly, the differences in sample collection and processing methods can also lead to variations in the results. In different studies, the tools used for collecting gingival crevicular fluid or periodontal pocket fluid, the collection sites, and the collection times may not be consistent. The differences in collection tools can affect the integrity of cells and biomolecules in the samples. The differences in collection sites can result in variations in the local inflammatory environment and the concentration of biomarkers. The differences in collection times may cause fluctuations in the results due to the time-dependent secretion of biomarkers. Moreover, the preservation conditions and processing methods of the samples, such as preservation temperature, centrifugation speed and time, and whether protective agents are added, can also affect the stability of biomarkers and the detection results. Furthermore, the differences in detection methods and techniques are also factors that cannot be ignored. Different studies may employ different detection techniques to measure the levels of biomarkers. These detection methods vary in terms of sensitivity, specificity, and accuracy, which may lead to inconsistent detection results for the same biomarker. Moreover, the performance of detection kits produced by different manufacturers may also differ, which will affect the accuracy and comparability of the detection results. Finally, the differences in the experimental design and quality control levels of the studies can also have an impact on the results. Some studies may not be rigorous in their experimental design, lacking sufficient control groups or having an insufficient sample size, which affects the reliability and stability of the results. In terms of quality control, different studies may have variations in the standardization of experimental operations, data recording, and analysis methods, all of which can lead to significant differences in the results among different studies.

This study has several limitations. First, in this meta-analysis, our aim was to systematically evaluate the association between smoking and periodontal inflammation and alveolar bone resorption in patients with chronic periodontitis. However, when analyzing the 10 biomarkers involved (such as ICAM-1, HMGB1, IL-17, PINP, NTX), it was found that only 2 to 3 studies were included for each outcome. The basic principle of meta-analysis is to reduce random errors by aggregating sufficient independent research data, thereby enhancing the reliability and generalizability of the results. However, the number of studies included in this research is relatively small, which may lead to certain biases in the results. Abnormal results from individual studies may have a significant impact on the aggregated results, making it difficult to accurately determine the true association between smoking and various biomarkers. Second, different studies vary in aspects such as research design, sample selection, data collection, and analysis methods, which may affect the accuracy and consistency of the research results. In terms of research design, some studies are prospective cohort studies, while others are retrospective case–control studies. Different study types may have differences in data collection and quality control. Prospective cohort studies can better determine causal relationships, but they may be affected by factors such as loss to follow-up; retrospective case–control studies may have issues such as recall bias. In terms of sample selection, different studies may include patients with differences in age, gender, race, and severity of the disease. Moreover, some studies may not have detailed descriptions of the inclusion and exclusion criteria for the samples, which may affect the representativeness and homogeneity of the samples. Third, in this study, significant to highly substantial heterogeneity was observed in almost all indicators. For instance, the I^2^ value for ICAM-1 was 97%, for IL-17 it was 96%, and for NTX it was 99%. The presence of heterogeneity greatly undermines the effectiveness of data aggregation and the credibility of the summarized results. The high degree of heterogeneity may imply that there are significant differences in the outcomes between different studies, and simply summarizing these results might lead to misleading conclusions. Although we are aware of the existence of heterogeneity, we have failed to fully explore its underlying causes. The sources of heterogeneity may involve multiple aspects. From the perspective of research design, different studies may have differences in inclusion and exclusion criteria, resulting in inconsistent severity of the patients’ conditions and thereby affecting the levels of biomarkers. Sample characteristics are also an important source of heterogeneity. There may be significant differences in the age distribution, gender ratio, racial composition, and smoking habits (such as smoking duration and daily smoking quantity) among different studies. Patients of different races have differences in their genetic background, which may lead to different levels of biomarkers. The differences in detection methods also cause heterogeneity. Different laboratories may use different reagents, instruments, and detection procedures when detecting biomarkers, and the operational norms and quality control during the detection process can also affect the accuracy of the results. Due to the failure to deeply analyze the causes of heterogeneity, we are unable to determine the authenticity and stability of the aggregated results. When interpreting the results of this study, we need to be cautious and should not directly generalize the aggregated results to all patients with chronic periodontitis. Fourth, this meta-analysis only included published literature, which may lead to the problem of publication bias. Studies with positive results are often more likely to be accepted and published by journals, while studies with negative results may have difficulty being published for various reasons (such as considering the results meaningless, fearing it would affect the journal’s reputation). This may result in the omission of some studies with negative results during the literature search, causing the summary results to be biased towards positive ones, thereby overestimating the strength of the association between smoking and periodontal inflammation and alveolar bone resorption in patients with chronic periodontitis. Although we conducted a comprehensive search using multiple databases and formulated strict inclusion and exclusion criteria, we still cannot completely rule out the possibility of publication bias.

Given the limitations of this study, such as the limited number of included studies and high heterogeneity, future research needs to further expand the sample size and scope. This can be achieved through multi-center collaboration, by including more patients with chronic periodontitis from different regions, races, and populations, to increase the representativeness and universality of the study. At the same time, the research design should be more rigorous and unified, with clear inclusion and exclusion criteria, standardized detection methods and operational procedures, and reduction of differences between studies. Furthermore, future research should also delve into the potential causes of heterogeneity. Through methods such as subgroup analysis and sensitivity analysis, the influence of different factors on the research results can be further examined. For instance, subgroup analysis can be conducted based on factors such as patients’ age, gender, race, and smoking habits to determine the differences in the relationship between smoking and periodontal health among different populations. Through these efforts, we hope to more accurately assess the association between smoking and periodontal inflammation and alveolar bone resorption in patients with chronic periodontitis, providing stronger evidence support for clinical practice.

In conclusion, this study demonstrates that during the development of periodontitis, there are excessive inflammatory responses in the periodontal tissues, which may subsequently lead to excessive absorption of alveolar bone. Specifically at the level of biomarkers, the study results show that the levels of ICAM-1, HMGB1 and IL-17 in the gingival crevicular fluid of the study group were significantly higher than those of the control group, while the level of YKL-40 showed no significant difference between the two groups. In terms of bone metabolism biomarkers, there was no significant difference in PINP and NTX levels between the two groups, but the level of CTX in the study group was significantly higher than that of the control group. Additionally, the level of OPG in the gingival crevicular fluid of the study group was significantly lower than that of the control group, while the levels of RANKL and MMP-9 were significantly higher than those of the control group. The differences in these biomarkers indicate that smoking not only exacerbates the inflammatory response in patients with periodontitis, but also disrupts the balance between bone-resorbing molecules and bone-protecting molecules, accelerating the process of alveolar bone absorption and thereby worsening the condition of periodontitis.

## Data Availability

The datasets presented in this study can be found in online repositories. The names of the repository/repositories and accession number(s) can be found in the article/supplementary material.
